# Editorial: Perturbations in Metabolic Cues: Implications for Adverse Cardiac Function Leading to Sudden Cardiac Death

**DOI:** 10.3389/fphys.2021.788904

**Published:** 2021-11-15

**Authors:** Brian P. Delisle, Ademuyiwa S. Aromolaran

**Affiliations:** ^1^Department of Physiology, University of Kentucky, Lexington, KY, United States; ^2^Department of Surgery, Division of Cardiothoracic Surgery, Nora Eccles Harrison Cardiovascular Research and Training Institute and Molecular Medicine Program, University of Utah School of Medicine, Salt Lake City, UT, United States

**Keywords:** arrhythmia, sudden cardiac death, diabetes, obesity, inflammation, metabolism, cardiomyocyte

Sudden cardiac death (SCD) remains a leading cause of death in the United States and accounts for ~460,000 deaths annually (Zheng et al., 2001; Roger et al., 2012). Importantly, there is increasing evidence that metabolic syndrome, a disease process defined by a clustering of pathologies (obesity, diabetes, dyslipidemia and inflammation), is a significant contributor to the risk for SCD (Yang et al., 2015). Therefore, it is important to understand the intertwined complexity of metabolic disorders and how cardiomyocytes respond to the associated electrical and structural perturbations. In order to develop novel and effective strategies to prevent SCD in people with metabolic disorders there is a need to identify metabolic risk factors. In this Research Topic we present a series of articles from experts in various aspects of metabolic disorders and heart disease. Our hope is that the research ideas shared in these articles will create new discussions around existing gaps in knowledge and help initiate future metabolic studies in cardiac health and disease.

The Research Topic starts with a review article from van Opbergen et al. that describes an important role for pathological mitochondrial function in the development of arrhythmogenic cardiomyopathy (ACM), an electrical disease mechanism that increases the risk for SCD, particularly in young people and athletes (Corrado et al., 2017). While impaired mitochondrial function has been widely studied in the context of arrhythmogenesis, how this occurs in ACM remains unclear. The authors highlight defective mitochondrial ATP production and altered redox regulation as key mechanisms that may increase vulnerability to ACM. Thus, ACM studies that incorporate mitochondrial biology are emphasized with the expectation for novel mechanistic insights for the development of targeted therapies in people living with ACM.

A similar perspective to van Opbergen et al. is provided by Snyder et al.. In their review article, they discuss a critical role for cardiac metabolism, particularly as it relates to the electrical and structural properties that define normal cardiac sinus rhythm. Furthermore, they raise the possibility that a metabolism-based therapeutic strategy may be a promising approach in people with heart disease. The review by Xu et al. aims to emphasize the critical link between impaired cardiac metabolism and SCD. They elegantly guide the reader through the nicotinamide adenine dinucleotide (NAD^+^) pathway and how its impaired function may contribute to SCD. The authors conclude with a convincing argument in favor of NAD^+^-boosting therapies in people living with cardiovascular disease.

In a continuing dialogue about ATP production, myocardial energy metabolism and cardiac contractility, the review by Greenwell et al. provides a comprehensive perspective into distinct metabolic triggers that underlie acquired or genetic cardiomyopathies. Taken together, these reviews offer an intriguing prospect of how existing, as well as emerging data, suggest that metabolic pathways are spatially and temporally intertwined at multiple levels. Therefore, future studies that consider potential outcomes due to multiple or compartmentalized pathologies are likely to be informative and shift current paradigms.

Impaired beat-to-beat Ca^2+^ dynamics predispose to fatal arrhythmias, leading to heart failure (HF) (Balke and Shorofsky, 1998; Mora et al., 2019), and two research articles on this topic provide crucial evidence for reduced expression of cardiac bridging integrator 1 (cBIN1), a membrane curvature protein that generates Ca^2+^ microdomains at the myocyte t-tubular system. First, Hitzeman et al. demonstrated a novel finding that a high cBIN1 score (or CS) is an important marker of pathologic cardiac remodeling and predicts a 1-year risk of cardiovascular events in ambulatory people who have HF with preserved ejection fraction. This conclusion is also supported by the findings of Li et al.. They revealed that, in a mouse model of pre-existing HF, exogenous cBIN1 limited HF progression, improved cardiac function, and increased survival by normalizing t-tubule Ca handling microdomains. These findings are clinically relevant and further underscore the importance of CS testing with the ability to detect smaller changes in t-tubule cBIN1 levels in pre-HF, especially those cases that are associated with metabolic disorder.

Two articles in this Research Topic focused on circadian mechanisms, arrhythmias, and risk for SCD. Bernardi et al. explores how the circadian-dependent modulation of metabolic and environmental cues, lead to altered hormonal and autonomic signaling and contribute to arrhythmias. Schroder et al. investigated how the disruption of the cardiac circadian clock by inducing the deletion of *Bmal1*, the core circadian clock transcription factor, impacts cardiac function in a knock-in mouse (*Scn5a*^+/Δ*KPQ*^) model of long QT syndrome type 3 (LQT3). The authors demonstrated that inducing the deletion of *Bmal1* in cardiomyocytes decreased the expression of several different cardiac ion channel mRNA transcripts and increased the QT interval at slow heart rates in wild-type and *Scn5a*^+/Δ*KPQ*^ mice.

The relationship between the metabolic syndrome and acquired arrhythmias was also studied by Yang et al. who investigated the effects of low magnesium (or hypomagnesemia) on electrocardiographic (ECG) parameters. Hypomagnesemia is acquired in people with metabolic disorders (Guerrero-Romero and Rodríguez-Morán, 2002), and here Yang et al. shows that people with hypomagnesemia displayed significant ECG changes, including an increase in the heart rate corrected QT interval and P wave duration, consistent with both impaired atrial and ventricular repolarization. Overall, these findings highlight how individual metabolic mechanisms may independently modify cardiac function.

The article by Espinoza et al. highlights the interplay between cardiometabolic function, parasympathetic (or vagal) motor output and the effects on cardiac function. Specifically, the authors focus their discussion on how vagal motor output may modulate metabolic cues and affect cardiac function. The authors state that vagal brainstem circuits provide a unique integrative mechanism that regulates and responds to metabolic cues to impact cardiac function.

Impaired thyroid function and the metabolic syndrome are interlinked endocrine disorders with significant morbidity and mortality in people worldwide. Additionally, thyroid hormone (TH) plays an important role in modulating cardiac electrophysiological properties. The article by Yamakawa et al. discusses the current knowledge on TH action on the cardiovascular system, relevant clinical and hemodynamic laboratory findings, and the therapeutic management for people with hyper- or hypo-thyroid heart disease. They also discuss cardiovascular medications that can disrupt normal thyroid function, including amiodarone-induced thyroid toxicity.

Overall, this Research Topic continues the discussion for how perturbations in several distinct metabolic mechanisms can have significant implications for cardiac function. Specifically, the articles published in this topic highlight how normal and impaired metabolic cues can impact electrical, structural, neural, and hormonal modulation of cardiac function. Expanding research that explores how metabolic disorders impact and contribute to heart disease will advance the field in ways that improve therapeutic options, better the quality of life for people living with heart disease and reduce the risk for SCD.

## Author Contributions

BD and ASA contributed significantly to this work, finalized, and approved it for publication. Both authors contributed to the article and approved the submitted version.

## Funding

This work was supported by the NIH (R01 HL147044 to ASA; R01HL153042 and R01HL141343 to BD).

## Conflict of Interest

The authors declare that the research was conducted in the absence of any commercial or financial relationships that could be construed as a potential conflict of interest.

## Publisher's Note

All claims expressed in this article are solely those of the authors and do not necessarily represent those of their affiliated organizations, or those of the publisher, the editors and the reviewers. Any product that may be evaluated in this article, or claim that may be made by its manufacturer, is not guaranteed or endorsed by the publisher.

## References

[B1] Balke C. W. Shorofsky S. R. (1998). Alterations in calcium handling in cardiac hypertrophy and heart failure. Cardiovasc. Res. 37, 290–299. 10.1016/S0008-6363(97)00272-1 9614486

[B2] Corrado D. Basso C. Judge D. P. (2017). Arrhythmogenic cardiomyopathy. Circul. Res. 121, 784–802. 10.1161/CIRCRESAHA.117.309345 28912183

[B3] Guerrero-Romero F. Rodríguez-Morán M. (2002). Low serum magnesium levels and metabolic syndrome. Acta Diabetol. 39, 209–213. 10.1007/s005920200036 12486495

[B4] Mora M. T. Gomez J. F. Morley G. Ferrero J. M. Trenor B. (2019). Mechanistic investigation of Ca2+ alternans in human heart failure and its modulation by fibroblasts. PLoS ONE 14:e0217993. 10.1371/journal.pone.0217993 31211790 PMC6581251

[B5] Roger V. L. Go A. S. Lloyd-Jones D. M. Benjamin E. J. Berry J. D. Borden W. B.. (2012). Heart disease and stroke statistics−2012 update. Circulation 125, e2–e220. 10.1161/CIR.0b013e3182456d46 22179539 PMC4440543

[B6] Yang K.-C. Kyle J. W. Makielski J. C. Dudley S. C. (2015). Mechanisms of sudden cardiac death. Circul. Res. 116, 1937–1955. 10.1161/CIRCRESAHA.116.304691 26044249 PMC4458707

[B7] Zheng Z.-J. Croft J. B. Giles W. H. Mensah G. A. (2001). Sudden cardiac death in the United States, 1989 to 1998. Circulation 104, 2158–2163. 10.1161/hc4301.098254 11684624

